# Correlation between chronic kidney disease and all-cause mortality in diabetic foot ulcers: evidence from the 1999-2004 national health and nutrition examination survey (NHANES)

**DOI:** 10.3389/fendo.2025.1533087

**Published:** 2025-03-14

**Authors:** Jiaru Liang, Hang An, Xuyang Hu, Yuling Gao, Jiaqi Zhou, Xiaoyang Gong, Junwei Zong, Yong Liu

**Affiliations:** ^1^ Department of Rehabilitation Medicine, First Affiliated Hospital, Dalian Medical University, Dalian, China; ^2^ College of Integrative Medicine, Dalian Medical University, Dalian, China; ^3^ Department of Orthopaedic Surgery, First Affiliated Hospital, Dalian Medical University, Dalian, China; ^4^ College of Health-Preservation and Wellness, Dalian Medical University, Dalian, China

**Keywords:** diabetic foot ulcers, all-cause mortality, NHANES, chronic kidney disease, cystatin C

## Abstract

**Background:**

The poor prognosis of diabetic foot ulcers (DFUs) often leads to amputation and high mortality rates, becoming a heavy economic burden on the healthcare system. Several clinical studies have been conducted to investigate the risk factors for DFU mortality and to provide clinical guidance for better prevention and control of DFU mortality.

**Methods:**

We used R to organize the mortality data of patients with DFU, collected from the NHANES database during the 1994-2004 period, along with three kidney function indicators including Albumin-to-Creatinine Ratio (ACR), estimated Glomerular Filtration Rate (eGFR) and cystatin C, used to assess chronic kidney disease (CKD). We explored the relationship between CKD and the risk of death in DFU patients through multiple kidney function indicators. Baseline characteristics of the surviving group and the mortality group of patients with DFU were analyzed using the ‘svyby’ function in the ‘survey’ package. We used Kaplan-Meier curves, multivariable logistic regression models, Cox proportional risk regression models, and time-dependent ROC curves to analyze the relationship between CKD and the risk of death in patients with DFU.

**Results:**

This study included a total of 112 patients with DFU. The overall sample had an average age of 65 years, with 43 females (38.39%) and 69 males (61.61%). During the follow-up time, 29 survived and 89 died. All-cause mortality in DFU patients was analyzed based on clinical classifications of ACR, eGFR, and cystatin C, with Kaplan-Meier curves illustrating survival variability. Multivariable logistic regression analysis showed no significant correlation between the risk of death in patients with DFU and CKD. However, analysis of Cox proportional risk regression model that accounted for time effects found a significant association between all-cause mortality and cystatin C levels in patients with DFU. Time-dependent ROC curve analysis demonstrated that cystatin C had superior diagnostic accuracy and stability for predicting all-cause mortality in DFU patients.

**Conclusions:**

In this study, we found that cystatin C demonstrated greater stability and accuracy in assessing the risk of death and predicting mortality in patients with DFU.

## 1 Introduction

Diabetic foot ulcers (DFUs) represent one of the most severe lower extremity complications of persons with diabetes, characterized by complex and prolonged etiology involving neurologic, vascular, and infectious mechanisms (1). Lihong Chen et al.’s meta-analysis, involving 34 studies and 124,376 participants, revealed high mortality rates in DFU patients: 13.1% at 1 year, 49.1% at 5 years, and 76.9% at 10 years (2). David G. Armstrong and colleagues collected data from literature published five years after 2007, showing a 5-year mortality rate of 30.5% for DFU. In the United States, compared to the research-focused cancer, the healthcare costs for DFU in 2017 were equivalent to the direct costs of cancer in 2015 (3).

Not only is there evidence that chronic kidney disease (CKD) is associated with an increased risk of foot ulcer in diabetic patients (4), but there are also certain clinical studies that have found CKD to be associated with mortality in patients with DFU. Infection, amputation, CKD, and improper care are common factors that increase the risk of death in patients with DFU (5). Valentina Guarnotta et al. collected mortality of DFU in Sicilian Type 2 diabetic patients hospitalized in 2008-2013 and 2014-2019, and found that only moderate-to-severe CKD, age of onset greater than 69 years, and eGFR lower than 92 were independently associated with risk of death (6). Aragón-Sánchez et al. found that albuminuria was a predictor of in-hospital mortality not only in diabetic patients but also in patients with FU in 455 patients (7).

Based on current research on CKD and the mortality rate of DFU patients, further analysis is needed to better evaluate the indicators related to CKD and mortality in DFU patients, as well as the death risk within them. Therefore, we collated mortality data for patients with DFU collected from the National Health and Nutrition Examination Survey (NHANES) population database during 1994-2004, as well as three indices of renal function, including albumin creatinine ratio (ACR), estimated glomerular filtration rate (eGFR), and cystatin C, which is used in the assessment of chronic kidney disease (CKD), to explore the relationship between CKD and the risk of death in patients with DFU. This analysis provides a basis for discussing the impact of CKD stage on the occurrence and prognosis of DFU-related mortality.

## 2 Materials and methods

### 2.1 Research subjects

We included DFU patients diagnosed with diabetes from the NHANES database between 1999 and 2004, who had a history of non-healing ulcers for more than four weeks according to the diabetes questionnaire data. Mortality follow-up data for this population was obtained from the National Death Index (NDI). Patients under the age of 40 and pregnant women were excluded based on the scope of the questionnaire.

### 2.2 Indicators of CKD

ACR, eGFR and cystatin C were used as indicators to assess CKD, and the calculations required serum creatinine, urinary creatinine, and urinary protein, where eGFR was calculated using the CKD-EPI formula (8). The CKD-EPI formula for creatinine values using mg/dL is specified as follows: eGFR = 141 × min(Scr/κ, 1)^α^ × max(Scr/κ, 1)^-1.209^ × 0.993^Age^ × 1.018 [if female] _ 1.159 [if black], where Scr is serum creatinine, κ is 0.7 for females and 0.9 for males, α is -0.329 for females and -0.411 for males, min indicates the minimum of Scr/κ or 1, and max indicates the maximum of Scr/κ or 1.

### 2.3 Covariates

Covariates potentially associated with DFU and likely to influence the association of DFU with CKD were included in this study. The multiple estimation of chained equations (MICE) method was used to estimate the missing covariate data. Diabetic patients included in the survey should contain any of the following conditions: (1) HbA1c ≥6.5%; (2) fasting blood glucose ≥7.0 mmol/L (≥ 126 mg/dL); (3) random blood glucose ≥11.1 mmol/L(≥ 200 mg/dL); and (4) had been diagnosed with diabetes by a physician. Biochemical indicators include total cholesterol (TC), serum albumin. Hypertension was defined as a diagnosis confirmed by a physician. Peripheral neuropathy (PN) was defined as having at least one insensate site in both feet. Peripheral arterial disease (PAD) was defined as a left ankle or right ankle brachial index (ABI) < 0.9. Body mass index (BMI) was obtained directly from body measurements. The current smokers were defined as participants who were reported smoking occasionally or daily during the past 7 days, or over 100 cigarettes.

### 2.4 Statistical analysis

NHANES data meeting the inclusion criteria were extracted, merged, filtered, and analyzed using weighted data in R Studio (version 4.4.1). For baseline characteristics, means and standard deviations were calculated for continuous variables in the deceased and non-deceased groups using the ‘svyby’ function from the ‘survey’ package, while percentages and 95% confidence intervals were calculated for categorical variables. We obtained p-values by fitting a generalized linear model (GLM) using the ‘svyglm’ function and calculating t-tests for the regression coefficients to assess whether there was a significant between-group difference between the surviving and dying groups.

**Figure 1:**
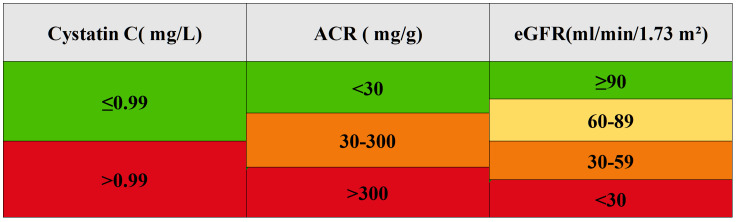
Grading chart of renal function indicators.

We transformed eGFR and ACR into categorical variables using clinical classification: eGFR was divided into four categories (≥90, 60-89, 30-59, <30 ml/min/1.73 m²), and ACR into three categories (<30, 30-300, >300 mg/g). The optimal cutoff value for cystatin C was determined using the cutoff in the R package, divided into two groups of ≤0.99 and >0.99 mg/L for analysis (**Figure 1**).

The Kaplan-Meier (KM) survival curves visually demonstrates the relationship between renal function indices at various clinical grades and the survival probability of DFU patients over time.

Using the ‘svyglm’ function and adjusting for confounding variables, a multivariable logistic regression analysis was conducted with a generalized linear model (GLM) to investigate the relationship between renal function indices at different clinical stages and the mortality risk in DFU patients. The odds ratio (OR) represents the change in mortality for each unit increase in renal function indices.

A Cox proportional hazards regression analysis was conducted to explore the relationship between CKD and the mortality risk in DFU patients. The hazard ratio (HR) was used to measure the impact of renal function on the mortality risk of DFU patients over time. The proportional risk assumption was tested using the Scheinfeld residual test. The proportional risk assumption was tested using the Scheinfeld residual test.

Using the ‘survivalROC’ package and based on survival data including survival time and status, the true positive rate (TPR) and false positive rate (FPR) are calculated using the Kaplan-Meier method to generate time-dependent ROC curves, while also computing the area under the curve (AUC) to quantify predictive performance. By traversing multiple time points, an AUC-time curve is plotted to analyze the trend of predictive accuracy over time, thus evaluating the long-term prognostic value of renal function indicators in the prognosis of DFU.

## 3 Results

### 3.1 Baseline characteristics of participants

**Figure 2:**
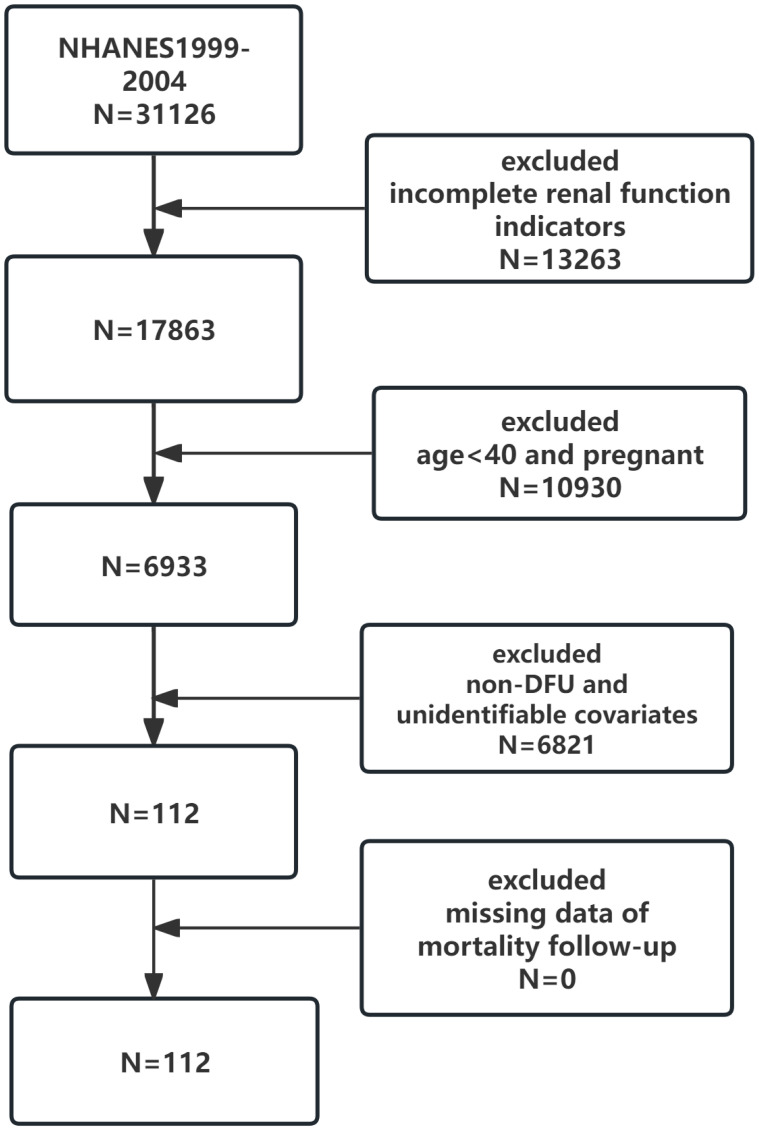
Flowchart of the participants selection.

A total of 31,126 people participated in the NHANES survey during the four cycles from 1999-2004. In our study, 10930 participants who were under 40 years of age and pregnant, 13263 with missing renal function data, 6790 non-DFU patients, and those who did not meet the diagnosis after data interpolation were excluded. Finally, 112 participants were included in our study, 29 in the surviving group and 83 in the mortality group (**Figure 2**).

The mean age of the overall sample was 65 years, of which 43 (38.39%) were females and 69 (61.61%) were males. Among the 112 DFU patients, the median follow-up duration was 116 months, with a mortality rate of 74.1%. The average age at the time of death was 63 years old. In our study, DFU mortality is significantly influenced by age, PAD, eGFR, and cystatin C levels. Older age (*P* < 0.01) and the presence of PAD (*P* < 0.001) are linked to higher mortality rates. Additionally, both eGFR (*P* < 0.05) and cystatinC (*P* < 0.01) indicate that patients with DFU and CKD face an elevated risk of death (**Table 1**).

**Table 1:** Basic characteristics of the participants in the surviving group and mortality group.

Characteristics	Surviving Group (n=29)	Mortality Group (n=83)	P-value
Age, years	53.66 ± 2.11	62.68 ± 1.85	<0.05
Gender, %			0.68
Male	64.51 (37.60,91.42)	58.79 (45.69,71.88)	
Female	35.49 (8.58,62.40)	41.21 (28.12,54.31)	
Race, %			0.92
White	71.16 (47.98,94.34)	72.48 (62.21,82.75)	
Other	28.84 (5.65,52.02)	27.52 (17.25,37.79)	
BMI, kg/m^2^	36.29 ± 1.84	33.72 ± 1.42	0.31
PAD, %			<0.0001
Yes	0.00 (0.00,0.00)	16.35 (6.59,26.11)	
No	100.00 (100.00,100.00)	83.65 (73.89,93.41)	
PN, %			0.30
Yes	33.05 (8.30,57.80)	49.75 (35.74,63.76)	
No	66.95 (42.20,91.69)	50.25 (36.24,64.26)	
Smoke, %			0.29
Yes	62.86 (35.89,89.82)	80.11 (67.74,92.49)	
No	37.14 (10.18,64.11)	19.89 (7.51,32.26)	
Hypertension, %			0.79
Yes	65.57 (39.01,92.12)	69.50 (57.26,81.73)	
No	34.43 (7.88,60.99)	30.50 (18.27,42.74)	
Total cholesterol, mmol/L	4.85 ± 0.24	5.07 ± 0.17	0.51
Serum albumin, g/dL	4.18 ± 0.11	4.03 ± 0.05	0.22
ACR, mg/g	183.00 ± 144.10	516.40 ± 206.20	0.20
eGFR, ml/min/1.73m^2^	85.19 ± 4.43	69.08 ± 3.62	<0.05
cystatin C, mg/L	0.87 ± 0.05	1.40 ± 0.17	<0.01

### 3.2 Correlation of CKD and mortality at DFU follow-up

**Figure 3:**
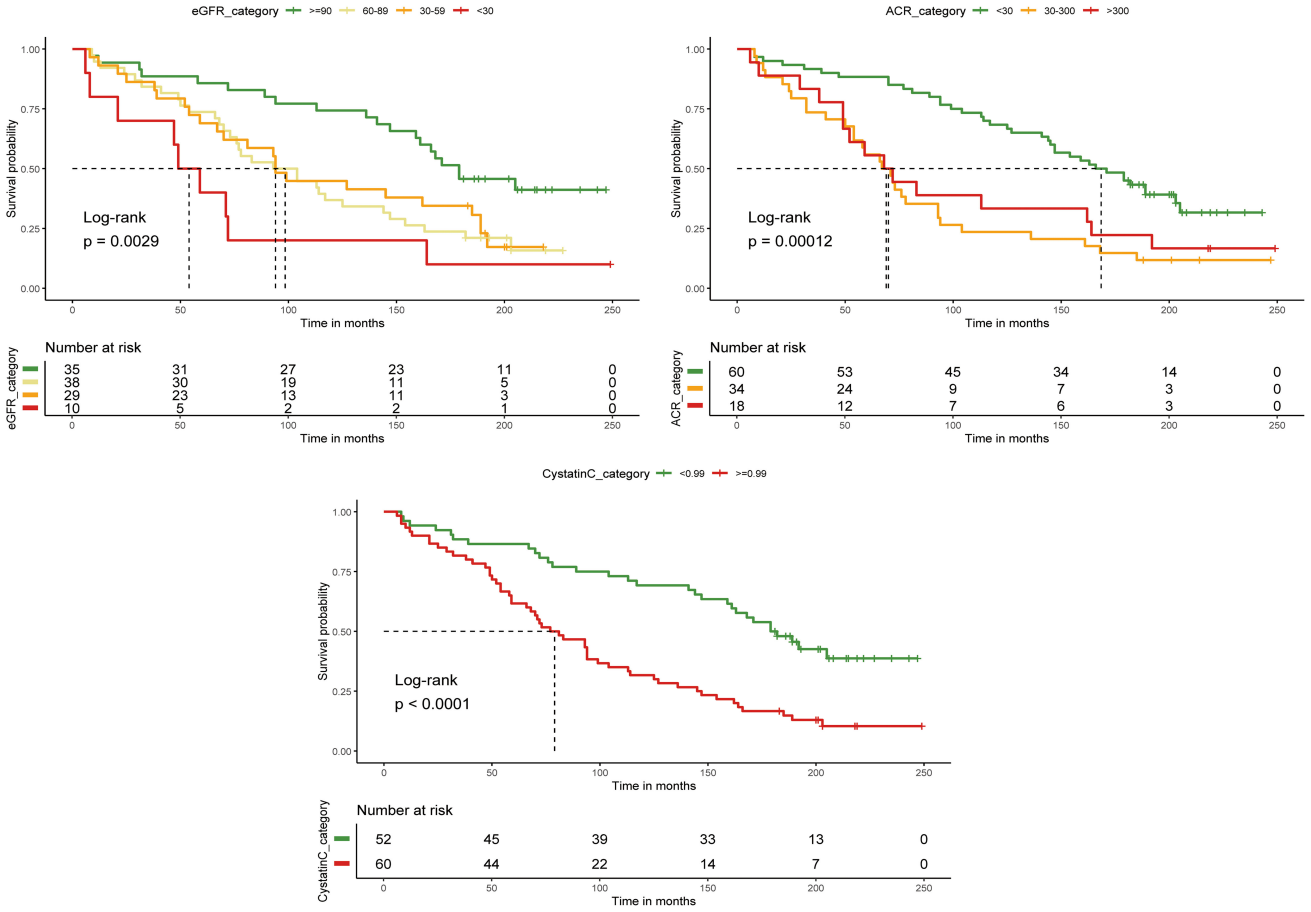
KM curves of the association between CKD and survival probability in DFU follow-up.

We converted cystatin C to a categorical variable using the optimal cutoff value, while eGFR and ACR were converted based on clinical guidelines. DFU patients with cystatin C levels > 0.99 mg/L had significantly shorter survival times compared to those with levels ≤ 0.99 mg/L. Similarly, patients with ACR < 30 mg/g had longer median survival times than those with ACR > 30 mg/g. However, no significant difference in survival was observed between patients with ACR > 300 mg/g and those with ACR between 30 and 300 mg/g. Patients with eGFR < 30 ml/min/1.73m² also exhibited significantly shorter survival times. These results were confirmed by the log-rank test (**Figure 3**).

### 3.3 The relationship between different categories of cystatin C, eGFR, and ACR with DFU mortality

In the multivariable logistic regression analysis, we constructed three models to gradually control for confounders in order to explore the relationship between CKD and the mortality risk of DFU. Model 1 did not adjust for any confounders, while Model 2 adjusted for basic demographic variables such as age, gender, and ethnicity. In these two models, CKD was significantly associated with the mortality risk of DFU patients, suggesting that CKD may have a direct impact on mortality risk. However, in Model 3, after further adjusting for potential confounders such as hypertension, peripheral artery disease, and smoking, the association between CKD and mortality risk was no longer significant. This indicates that the effect of CKD on mortality risk may be indirect, potentially mediated through these related comorbidities (e.g., hypertension, PAD) rather than CKD itself directly increasing the risk of death (**Table 2**).

**Table 2:** Three models for assessing the relationship between CKD and all-cause mortality in DFU.

		OR (95%CI)	P-value			OR (95%CI)	P-value			OR (95%CI)	P-value
ACR	Model 1			eGFR	Model 1			Cystatin C	Model 1		
	<30	Reference			≥90	Reference			≤0.99	Reference	
	30-300	10.45(1.88,58.07)	<0.05		60-89	1.64(0.29,9.37)	0.55		>0.99	7.29(2.01,26.49)	<0.05
	>300	8.71(1.34,56.68)	<0.05		30-59	2.25(0.35,14.46)	0.37				
					<30	61.29(4.78,786.56)	<0.05				
ACR	Model 2			eGFR	Model 2			Cystatin C	Model 2		
	<30	Reference			≥90	Reference			≤0.99	Reference	
	30-300	7.49(0.90,62.57)	0.06		60-89	1.36(0.18,10.30)	0.74		>0.99	5.34(1.35, 21.14)	<0.05
	>300	12.32(1.64,92.73)	<0.05		30-59	0.80(0.10,6.20)	0.81				
					<30	46.17(2.34,908.67)	<0.05				
ACR	Model 3			eGFR	Model 3			Cystatin C	Model 3		
	<30	Reference			≥90	Reference			≤0.99	Reference	
	30-300	5.81(0.09,378.76)	0.31		60-89	1.00(0.09,10.89)	0.10		>0.99	8.43(0.63,113.23)	0.09
	>300	6.52(0.17,244.45)	0.22		30-59	0.35(0.01,21.91)	0.48				
					<30	4.96(0.08,324.07)	0.31				

Model 1 did not adjust for adjustment confounders.

Model 2 adjusts for the following confounders: age, gender and race.

Model 3 adjusted for age, gender, race, hypertension, Serum albumin, TC, BMI, PAD, PN and smoke status.

### 3.4 Weighted Cox proportional hazards regression analysis and testing

**Figure 4:**
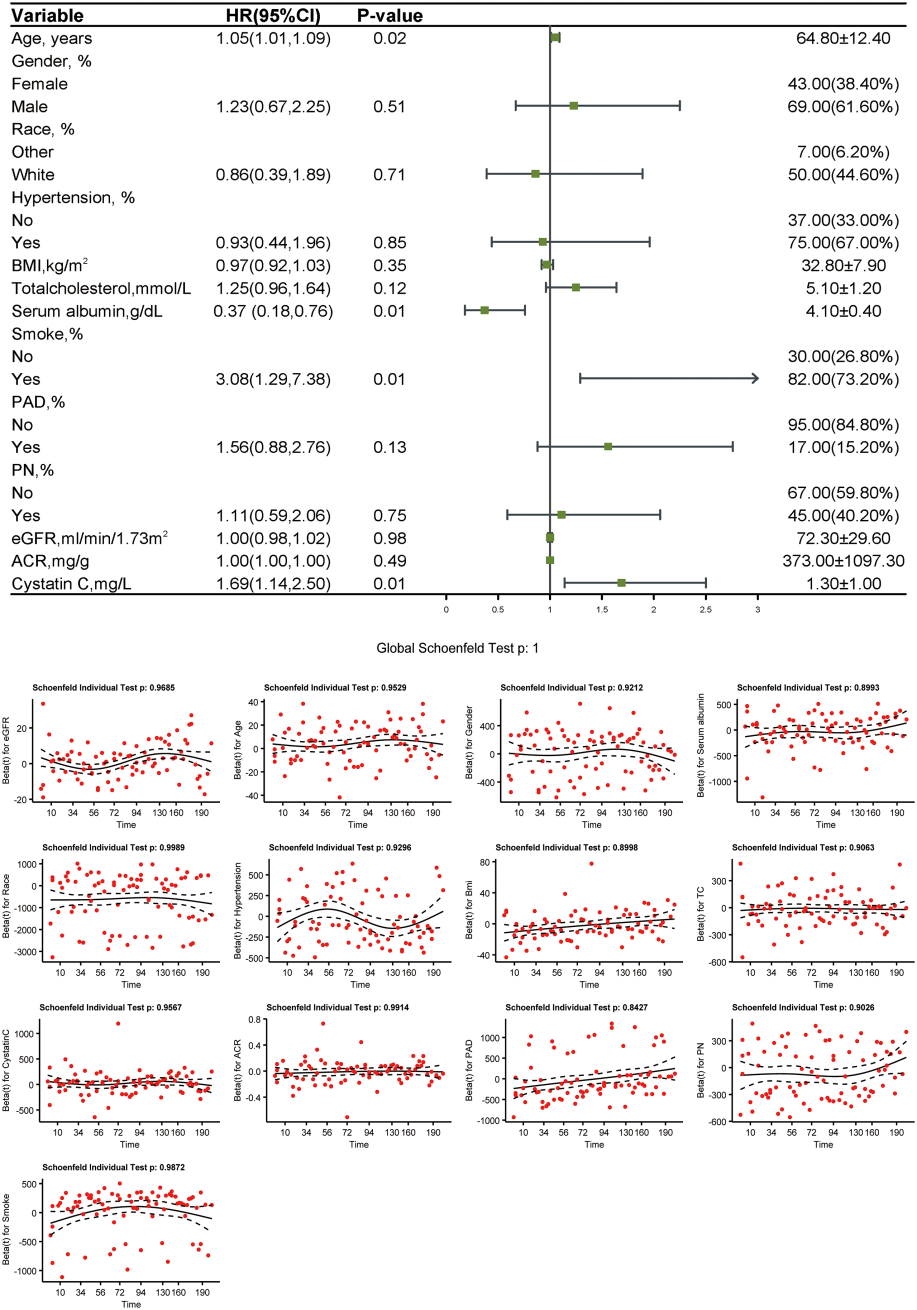
Forest plot of Cox proportional hazards regression analysis.

In the weighted Cox proportional hazards regression analysis, age (HR 1.05 [95% CI 1.01,1.09], *P* = 0.021), serum albumin [0.37(0.18,0.76), *P* = 0.007], smoking [3.08(1.29,7.38), *P* = 0.012], and cystatin C [1.69(1.14,2.50), *P* = 0.012] were significantly associated with mortality in patients with DFU. This suggests that these factors contribute to increased mortality risk due to their long-term cumulative effects on the body, which are more accurately captured in a time-dependent model like cox regression, compared to a static linear analysis. Schoenfeld residuals were used to test the proportional hazards assumption, and no violation was observed (**Figure 4**).

### 3.5 Time-dependent ROC curve analysis

**Figure 5:**
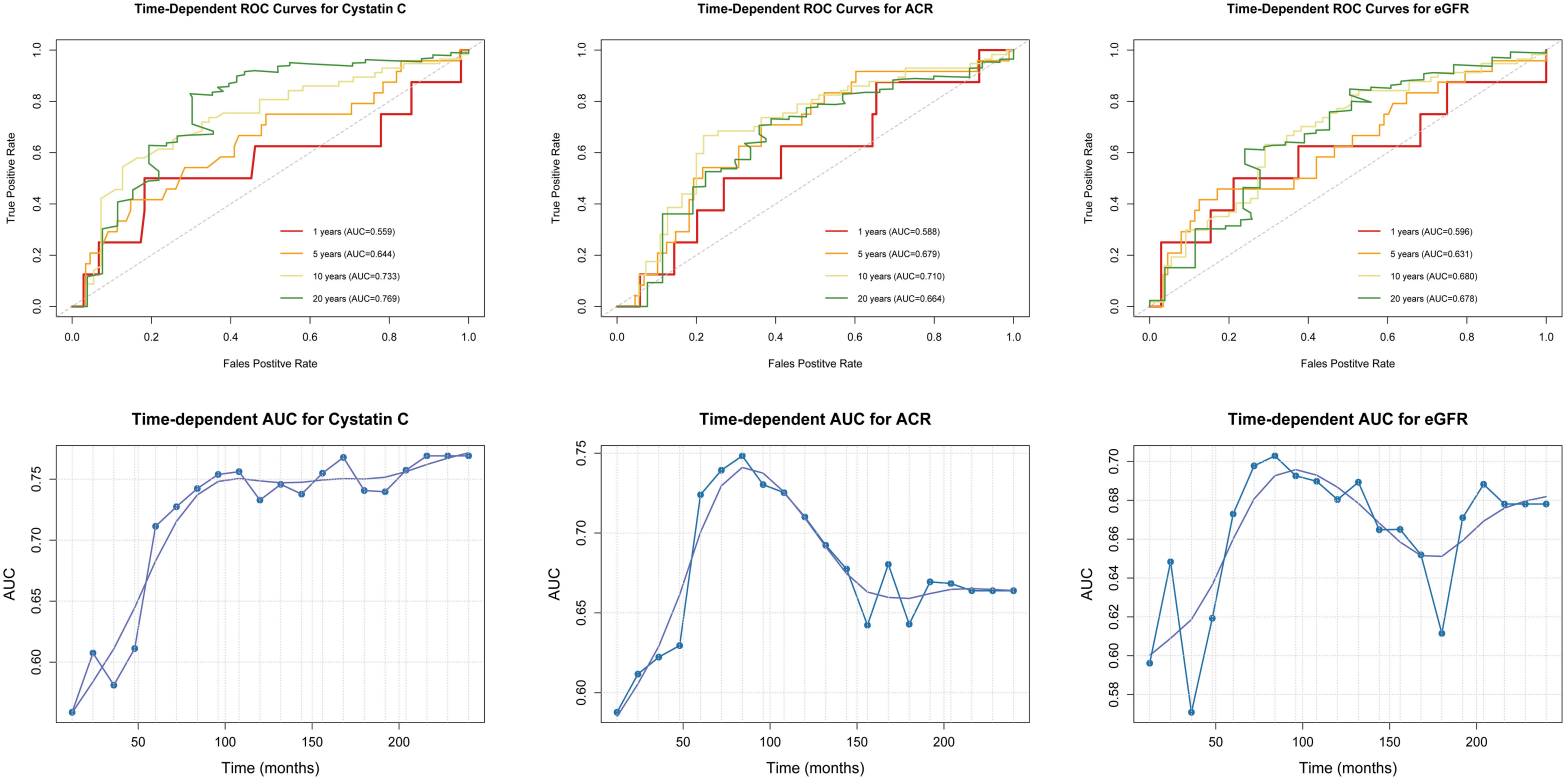
Time-dependent ROC curves and AUC-time curves for the association between CKD and mortality risk in DFU.

The time-dependent ROC curve analysis indicates that the correlation between cystatin C and mortality outcomes in patients with DFU increased steadily over time, whereas the correlation between ACR and eGFR decreased after 10 years. We plotted AUC-time curves in months at 12-month intervals to further show that the close correlation between cystatin C and the risk of death in patients with DFU remained stable after 5 years of follow-up (AUC>0.70), whereas the correlation between ACR and eGFR and the risk of death from DFU declined after 7 years of follow-up, with the AUC value dropping below 0.70 after 10 years.(**Figure 5**).

## 4 Discussion

As the overall incidence of diabetes and the life expectancy of diabetic patients increase, the likelihood of developing DFU rises with the chronic nature of the disease (9). DFU impose a substantial burden on healthcare systems, and effective management of patients with DFU requires the support of a multidisciplinary team. DFU contributes significantly to healthcare costs; in 2017, the direct costs of diabetes complications in the U.S. were estimated at $237 billion, with approximately 33% of that attributed to DFU (10).Current studies have identified several factors associated with DFU mortality. Demographically, older age (11) and male gender (12) are significant risk factors. In terms of complications, DFU patients with Cardiovascular disease (CVD) (13), PAD (14) and CKD (15) have a much higher mortality rate. End-stage renal disease (ESRD) and CKD are associated with a higher risk of DFU events, longer healing times, higher ulcer recurrence rates, and increased rates of lower extremity amputations.

In our study, we first analyzed the follow-up survival and mortality groups of patients with DFU, which were differentiated by the mean and percentage of age, PAD, eGFR, and cystatin C. Then, we visualized the survival probability of CKD at different time points by KM curves, and we also compared the difference in the survival probability of different renal function indicators. DFU patients with eGFR < 30 ml/min/1.73m^2^ had significantly reduced survival times, with less than 5 years of survival. The optimal cutoff value for cystatin C was 0.99 mg/L and there was significant variability in survival. Multivariable logistic regression model analysis revealed that the three renal function indicators were not significantly associated with the risk of death in patients with DFU at different grades. We also chose the Cox proportional risk regression model for the analysis of mortality risk in patients with DFU and CKD, and found that an increase in cystatin C was a significant correlation with an increased risk of death in patients with DFU. Finally, we assessed the long-term predictive value of renal function indicators in DFU mortality by time-dependent roc curves and AUC-time curves and found that cystatin C exhibited greater accuracy than ACR and eGFR in predicting mortality beyond five years for DFU patients and maintained its predictive accuracy over time. We verified the correlation between CKD and risk of death in patients with DFU, compared the correlation between renal function indicators and risk of death in patients with DFU and attempted to carry out the predictive ability of renal function indicators for death in patients with DFU.

Diabetic patients are in a prolonged state of hyperglycemia generating oxidative stress and pro-inflammatory responses affecting organ systems throughout the body, leading to nerve damage, microvascular disease, macrovascular damage, and the formation of multiple complications (16). Diabetic nephropathy (DN) and DFU are the same major complications involving nerves, microvessels and macrovessels (17).CKD may result in an increased risk of death in patients with DFU from the following mechanisms: 1.Nerve damage: CKD-induced retention of potassium ions and metabolites such as creatinine and urea increases peripheral nerve damage impairing sensation and muscle function in the distal lower extremities (18). 2.Vascular damage: fluid retention, renin-angiotensin system changes, oxidative stress resulting in more serious peripheral vascular disease aggravated ischemic ulcers (19). 3. Poor nutritional status: metabolic disorders leading to loss of micronutrients, increased renal filtration of proteins leading to hypoalbuminemia, and anemia due to relative EPO deficiency (20).

Cystatin C, as an indicator of kidney function, is commonly used to predict mortality in populations such as elderly males (21), patients with CVD (22), and individuals with diabetes (23). Compared to serum creatinine, which is easily influenced by age and muscle mass, cystatin C can independently reflect kidney function (24). Also cystatin C is closely related to sarcopenia. In a study by Qin Yang et al, sarcopenia was found to be an independent risk factor for all-cause mortality in patients with DFU, and therefore an important prognostic factor for patients with DFU (25), and sarcopenia is one of the poor prognostic factors for CKD (26).In the study of mortality outcomes in patients with DFU, cystatin C is a potential predictive marker in patients with DFU with a composite chronic kidney disease condition or independently reflecting an individual’s adverse muscular condition.

## 5 Conclusion

Our study is based on participants from the NHANES database from 1999 to 2004 and their follow-up mortality data. We compared the correlation between different kidney function indicators, including cystatin C, ACR, and eGFR, with mortality in DFU patients, as well as their diagnostic accuracy for mortality. Compared to ACR and eGFR, cystatin C demonstrated greater stability and accuracy in assessing the risk of death and predicting mortality in patients with DFU.

## 6 Study limitation

There are some shortcomings in our study: the foot ulcer data in the NHANES database was only investigated from 1999 to 2004 and only for those aged 40 years and over, resulting in a small sample size and fewer covariates to include in our analysis. There is a delay in analyzing recent disease conditions because the data are old and not updated.

## Data Availability

The original contributions presented in the study are included in the article/supplementary material. Further inquiries can be directed to the corresponding authors. The data included in this study can be accessed directly from the link in **Table 3**. Web links to data.
